# Bumbling, Stumbling, Fumbling: Weakness, Steppage Gait, and Facial Droop in a 3-Year-Old Male

**DOI:** 10.1177/2333794X19865858

**Published:** 2019-07-24

**Authors:** Aviva J. Whelan, Abdullah Tolaymat, Shane C. Rainey

**Affiliations:** 1 University of Illinois College of Medicine at Peoria, Peoria, IL, USA

**Keywords:** Guillain-Barré Syndrome, neurology, pediatrics

## Abstract

A previously healthy, unimmunized, 3-year-old Caucasian boy presented to the emergency department with right-sided facial droop, clumsiness, and intermittent bilateral hip pain. Two weeks ago, he had 24 hours of self-resolving rhinorrhea and fever. Examination was significant for right facial nerve palsy, lower extremity pain, areflexia of his right lower extremity, and diminished reflexes of his left lower extremity. He was admitted for urgent magnetic resonance imaging of the brain. Cerebrospinal fluid (CSF) protein was 85 mg/dL with elevated albumin and immunoglobulin, and CSF white blood cell was 3 cells/mm^3^. Serum *Mycoplasma* immunoglobulin (Ig) M and IgG were elevated. There was concern for Guillain-Barré syndrome (GBS). He was started on intravenous IG (IVIG) and was treated for presumed *Mycoplasma* infection. Weakness and gait disturbances in a child can present the clinician with a diagnostic challenge. Gait disturbance may indicate a neurological lesion anywhere from the central nervous system to the peripheral nerves, neuromuscular junction, or muscle. In the present case, the combination of peripheral facial palsy, presumed neuropathic pain, gait difficulties, and areflexia in the setting of an antecedent respiratory illness were suggestive of GBS. The cornerstone treatments involve hospitalization to facilitate continuous monitoring for serious sequelae, such as acute respiratory failure and cardiac dysrhythmia, followed by immunotherapy with IVIG or plasma exchange. Gait disturbance and weakness in a child is a diagnostic challenge. GBS is the most common cause of acute paralysis in the Western world and should remain high on the clinician’s differential diagnosis. However, patients with GBS may also present nonclassically with extremity pain and cranial nerve palsies.

## Case Report

A previously healthy, unimmunized, 3-year-old Caucasian boy presented to the emergency department (ED) with progressively worsening right-sided facial droop and intermittent bilateral hip pain. His parents reported that over the past week, he appeared to be clumsy and was stumbling frequently. Approximately 3 days prior to presentation, mother reported that the patient developed right-sided facial droop, hoarseness, and intermittent bilateral hip pain that occurred mainly with dressing and hip flexion. He was evaluated by his pediatrician, where a complete blood count, comprehensive metabolic profile, and creatinine kinase were normal and recommended close observation with instructions to return with worsening symptoms. Over the next 2 days, the patient fell twice, had worsening facial droop, and developed progressive dysphonia, prompting his parents to take him to the ED. He did not have headache, dyspnea, otalgia, sore throat, abdominal pain, nausea, vomiting, diarrhea, or skin rash. Parents stated that 2 weeks ago, he had 24 hours of self-resolving rhinorrhea and fever of 101°F for which they did not seek medical attention. They also reported that they recently visited their cabin in the “woods” but deny any known tick bites.

In the ED, he was afebrile with a heart rate of 107 beats per minute, a respiratory rate of 28 breaths per minute, a blood pressure of 109/70 mm Hg, and an oxygen saturation of 100% on room air. Physical examination showed an ill-appearing child in no acute distress. His neurological examination was significant for pronounced right facial nerve palsy with inability to close his right eye or wrinkle his right forehead. His right nasolabial fold was flattened, and he had an asymmetric frown. He had pain with hip flexion bilaterally and bilateral foot drop more noticeable with steppage gait. Strength in the proximal lower extremities at the quadriceps, hamstrings, and gastrocnemius was 5/5. Anterior tibialis strength was 3/5 bilaterally. There was concomitant areflexia of his right patellar and Achilles reflexes and 1 out of 4 left patellar and Achilles reflexes. Babinski reflex was down going bilaterally, and there was no clonus. Sensory examination was intact. Laboratory studies including complete blood count, comprehensive metabolic profile, magnesium, phosphorus, erythrocyte sedimentation rate, C-reactive protein, and a urine drug screen were within normal limits. He was admitted for urgent neurologic and infectious evaluation.

## Hospital Course

The patient underwent urgent evaluation with magnetic resonance imaging of his brain, lumbosacral spine, and sacral plexus, which was negative for space occupying lesions and vascular pathology. A lumbar puncture revealed a cerebrospinal fluid (CSF) protein of 85 mg/dL, a white blood cell count of 3 cells/mm^3^, and no organisms. CSF albumin and IgG levels were subsequently found to be elevated as well. A serum *Mycoplasma* immunoglobulin (Ig) M and IgG were elevated. The remainder of his CSF studies including enterovirus polymerase chain reaction (PCR), herpes PCR, *Cryptococcus* antigen, oligoclonal bands, ganglioside antibodies, Lyme antibodies, and *Mycoplasma* PCR were negative. Additional infectious workup included a QuantiFERON assay for *Mycobacterium tuberculosis* and antibodies to West Nile virus, which were both negative.

Given the combination of ascending areflexia and pain, CSF albuminocytologic dissociation, and a remote upper respiratory tract infection, there was concern for acute inflammatory demyelinating polyneuropathy (AIDP). He was admitted to the inpatient ward, placed on continuous telemetry monitoring, had bedside spirometry, and started on intravenous IG (IVIG) at 2 g/kg, which was given over 3 days. An electrocardiogram was notable for intermittent premature ventricular contractions without significant dysrhythmia. Electromyography (EMG) was not performed. A swallowing evaluation was completed due to dysphonia and facial nerve palsy and was negative for frank aspiration or deep laryngeal penetration. He was treated for presumed *Mycoplasma* infection with a 5-day course of azithromycin. After completing IVIG, his lower extremity strength and ambulatory abilities slowly began to improve over the course of the next week with the assistance of physical therapy. He was started on gabapentin for suspected neuropathic pain, which his parents report greatly facilitated his work with physical therapy. After 7 days, the patient was able to ambulate independently, had no signs of bulbar weakness, autonomic instability, or respiratory insufficiency, and was subsequently discharged. Thereafter, he was closely followed in the outpatient neurology clinic, where he continued to demonstrate remarkable improvement including normal walking and return of reflexes.

## Final Diagnosis

Acute inflammatory demyelinating polyneuropathy, the most common form of Guillain-Barré syndrome (GBS), likely secondary to *Mycoplasma pneumoniae* infection.

## Discussion

Weakness and gait disturbances in a child can present the clinician with a diagnostic challenge, as the differential diagnosis is exceedingly broad. Gait disturbance may indicate a neurological lesion anywhere from the central nervous system to the peripheral nerves, neuromuscular junction, or muscle. Considerations include pathologies such as acute cerebellar ataxia, stroke, transverse myelitis, space-occupying spinal cord lesions, toxic neuropathy, tick paralysis, botulism, myasthenia gravis, polymyositis, and viral myositis, to name a few.^1^ While the subacute presentation of facial palsy and unilateral areflexia are not frequently associated with GBS, their combination with presumed neuropathic pain, gait difficulties, and ascending weakness in the setting of an antecedent respiratory illness were suggestive of GBS. Poliomyelitis and tick paralysis remained high on our differential given the patient’s unimmunized status and recent travel to the woods, although we suspected that *Mycoplasma* was the causative organism considering the known association with GBS and the patient’s total clinical picture.

Acute inflammatory demyelinating polyneuropathy is the most common type of GBS, which is an acute, postinfectious, inflammatory polyneuropathy that can manifest under several different phenotypic forms. Most commonly, GBS presents as an acute demyelinating polyneuropathy, which may involve various elements of the nervous system including spinal nerve roots, peripheral, and cranial nerves. There are several different variants of GBS, including AIDP, as well as the Miller-Fisher syndrome (characterized by ataxia, areflexia, and ophthalmoplegia), and polyneuritis cranialis. Many of these variants are associated with specific ganglioside antibodies that can be isolated from the CSF.^2^ Anti-GQ1b antibody, for example, is elevated in more than 80% of patients with Miller-Fisher syndrome. Furthermore, anti-GD1a and anti-GD1b antibodies are found in other GBS variants, such as acute motor axonal neuropathy and sensory ataxic neuropathy syndrome, respectively.^2^ In our case, these antibodies were sent and came back negative.

Since the eradication of poliovirus in the Western world, GBS is now the most common cause of acute paralysis in otherwise healthy children.^1,3^ The pathogenesis of GBS is incompletely understood, although there is compelling evidence to support an autoimmune-mediated mechanism.^4^ The clinical syndrome is classically observed several weeks after an antecedent diarrheal or respiratory illness, with *Campylobacter jejuni* most commonly implicated.^5,6^ Other ordinary culprits include cytomegalovirus, Epstein-Barr virus, and *Mycoplasma pneumoniae*.^4,5,7,8^

Clinically, GBS is characterized by a rapidly progressive, symmetric, ascending flaccid paralysis with concomitant areflexia. This ascending weakness may quickly evolve in severe cases to include the cranial nerves and diaphragm, a potentially fatal complication without prompt medical intervention.^1,9^ Symptoms generally range from lower back and leg pain to gait difficulties and refusal to bear weight, and cranial nerve involvement is typically demonstrated by bilateral facial weakness.^10,11^ Autonomic dysfunction is also commonplace and may manifest with cardiac dysrhythmia, orthostatic hypotension, ileus, or bladder dysfunction.^11,12^

Guillain-Barré syndrome is a clinical diagnosis, although several laboratory and radiographic investigations provide supporting evidence in the appropriate clinical context. CSF studies classically reveal an albuminocytologic dissociation, consisting of an elevated CSF protein without pleocytosis.^3,5,9^ EMG and nerve conduction studies are not frequently performed but are sensitive for diagnosis of GBS. EMG may demonstrate conduction abnormalities consistent with a demyelinating process. However, studies may be normal in acute cases, a normal study does not rule out GBS.^13^ Magnetic resonance imaging may show evidence of enhancement in the cauda equina or spinal nerve roots.^14^

The cornerstone treatments of GBS involve hospitalization to facilitate continuous monitoring for serious sequelae, such as acute respiratory failure and cardiac dysrhythmia, followed by immunotherapy with IVIG or plasma exchange.^3,5^ Clinicians should maintain a low threshold for delivering care in an intensive care unit, as approximately 10% to 20% of children with GBS will require mechanical ventilation.^1,15^ Indications for intensive care unit–level care include flaccid quadriparesis, reduced vital capacity, bulbar weakness, and autonomic instability.^16^ IVIG is not recommended for mild, nonprogressive cases of GBS as it does not correlate with improved outcomes, and it is expensive with potential for side effects (although overall minor). Mild patients are generally classified as having no impairment in walking, whereas severe patients are unable to walk. IVIG has shown to improve patients in severe disease, and repeated doses can be beneficial in severe, unresponsive GBS patients.^17^ IVIG is typically given in a dose of 2 g/kg divided over 3 to 5 days and is more frequently utilized over plasma exchange in children secondary to its availability, safety, and ease of administration. Several small studies have suggested that IVIG allows for more rapid neurological recovery times in comparison with supportive care alone.^18-20^ Therapeutic plasma exchange can also be used in cases of GBS. Whereas IVIG works by neutralizing harmful antibodies, plasmapheresis works by filtering antibodies out of the bloodstream. There is no indication to do both IVIG and plasmapheresis; Chevret et al found them to be equally effective.^21^

Overall, the prognosis of GBS in children is excellent when compared with the disease course in adults. Mortality is estimated at 3% to 4%, mostly due to respiratory failure or cardiac dysrhythmia.^1,11^ Long-term recovery rates are similarly encouraging. Approximately 85% of children will emerge from the disease either free of symptoms or without significant disability, and roughly 88% of children will regain ambulatory abilities within 6 months of their diagnosis.^22^

## Conclusions

Gait disturbance and weakness in a child is a diagnostic challenge. Patients with GBS may present nonclassically with areflexia and cranial nerve palsies. Prompt diagnosis and treatment are imperative to foster favorable clinical outcomes, especially given the potentially grievous complications. Increased recognition of GBS and efficient care have yielded an excellent prognosis for those children affected by an otherwise devastating disease. GBS is now the most common cause of acute paralysis in the Western world and should remain high on the clinician’s differential diagnosis of the child presenting atypically with areflexia, acute weakness, and gait disturbance.
